# Predominance of HBV genotype D in southern part of India

**DOI:** 10.1186/1471-2334-14-S3-P22

**Published:** 2014-05-27

**Authors:** Kalyanaraman Narayanan, L Thayumanavan, Jayalakshmi Mariakuttikan

**Affiliations:** 1 Department of Immunology, School of Biological Sciences, Madurai Kamaraj University, Madurai, India; 2 Department of Gastroenterology, Govt Rajaji Hospital, Madurai, India

## Background

The clinical outcome of HBV infection is highly heterogeneous which correlates with viral factors such as genotypes, viremia and mutants. Evidences showed that HBV genotypes have a role in prevalence of variants, IFN therapy and disease severity. So far, 10 HBV genotypes (A to J) have been identified with distinct geographical distribution. Hence, knowledge of the genotype infecting an individual may assist a physician making a decision towards better clinical management. The aim of this study is to identify the circulating HBV genotypes (A e) and its correlation with clinical manifestation.

## Methods

A cohort of 72 patients (Acute: 11; Asymptomatic: 36; Chronic: 24; and HCC: 1) attending the Govt. Rajaji Hospital, Madurai, was recruited. Clinical categorization was based on biochemical and viral markers. Viral DNA extraction was carried out from serum samples and genotyping was done by multiplex PCR.

## Results

Multiplex PCR was optimized with viral reference samples. Each reaction was carried out with five pairs of primers. We could observe only genotype D in this cohort (100%). None of the samples were assigned with other genotypes.

## Conclusion

HBV genotype D has a major role in emergence of precore mutants (1896 G>A) leading to diagnostic failure, vaccine escape mutants, and poor clinical response while therapy.

